# The Role of Autophagy for the Regeneration of the Aging Liver

**DOI:** 10.3390/ijms21103606

**Published:** 2020-05-20

**Authors:** Fengming Xu, Chuanfeng Hua, Hans-Michael Tautenhahn, Olaf Dirsch, Uta Dahmen

**Affiliations:** 1 Department of General, Visceral and Vascular Surgery, Jena University Hospital, 07747 Jena, Germany; Fengming.Xu@med.uni-jena.de (F.X.); Chuanfeng.Hua@med.uni-jena.de (C.H.); Hans-Michael.Tautenhahn@med.uni-jena.de (H.-M.T.); 2 Institute of Pathology, Klinikum Chemnitz gGmbH, 09111 Chemnitz, Germany; Olaf.Dirsch@gmail.com

**Keywords:** mTOR, AMPK, ULK1, TFEB, hepatectomy, hepatocyte, proliferation

## Abstract

Age is one of the key risk factors to develop malignant diseases leading to a high incidence of hepatic tumors in the elderly population. The only curative treatment for hepatic tumors is surgical removal, which initiates liver regeneration. However, liver regeneration is impaired with aging, leading to an increased surgical risk for the elderly patient. Due to the increased risk, those patients are potentially excluded from curative surgery. Aging impairs autophagy via lipofuscin accumulation and inhibition of autophagosome formation. Autophagy is a recycling mechanism for eukaryotic cells to maintain homeostasis. Its principal function is to degrade endogenous bio-macromolecules for recycling cellular substances. A number of recent studies have shown that the reduced regenerative capacity of the aged remnant liver can be restored by promoting autophagy. Autophagy can be activated via multiple mTOR-dependent and mTOR-independent pathways. However, inducing autophagy through the mTOR-dependent pathway alone severely impairs liver regeneration. In contrast, recent observations suggest that inducing autophagy via mTOR-independent pathways might be promising in promoting liver regeneration. Conclusion: Activation of autophagy via an mTOR-independent autophagy inducer is a potential therapy for promoting liver regeneration, especially in the elderly patients at risk.

## 1. Introduction

### 1.1. Aging Increases the Incidence of Malignancies

With the advancement of the health system, the lifespan of the population has increased significantly compared with the 1950s [1]. The incidence of cancer, an age-associated malignant transformation of cells, increased in parallel. Despite all developments, cancer-related mortality remains high. According to 2018 Global cancer statistics, the mortality of hepatic carcinoma was ranked second among various tumors in males [2].

### 1.2. Aging Increases the Risk of Liver Resection Due to Impaired Liver Regeneration

Aging in the human liver is causing morphological and physiological alterations, such as a decrease in liver volume and liver blood flow. Nonetheless, the aging liver can still maintain relatively normal metabolic functions under physiological conditions [3,4]. However, the regenerative capacity of the aging liver is significantly reduced compared with the young liver [5,6]. This age-related change has brought a dilemma to the clinical treatment of liver tumors in this subgroup of patients.

Liver resection and especially, extended resection is only performed in patients without obvious liver dysfunction, without severe dysfunction of other organs and without obvious impairment of liver regeneration. However, the mitotic capacity of the hepatocytes gradually declines with aging, leading to an impairment of liver regeneration in the elderly [6,7]. This impairment represents an additional risk for elderly patients, thereby possibly excluding these patients from curative surgery.

Patients with non-alcoholic fatty liver disease (NAFLD) or alcoholic cirrhosis have obviously impaired hepatic regenerative capacity [8,9]. Aging is often associated with similar morphological changes such as increased liver steatosis and fibrosis [10], which further limits the functional capacity of the aged remnant liver.

### 1.3. Aging Impairs Autophagy

Autophagy is an essential recycling mechanism of cellular components [11], which plays a crucial role in maintaining liver metabolism and also in promoting liver regeneration. Autophagy provides glucose, amino acids and free fatty acids to hepatocytes for maintaining their basal metabolic function [12]. In liver-specific *Atg5* knockout (KO) mice, liver regeneration was substantially declined with an impaired postoperative mitotic capacity after 70% partial hepatectomy (PH) compared with control animals. In the regenerating liver of the KO-mice, the ability to reorganize damaged proteins and organelles during autophagy was diminished [13].

Recently, several studies indicated that autophagy is substantially lower in the aged liver compared to the young liver [5,14,15,16]. Aging leads to a decrease in the number and function of autophagosomes and causes lipofuscin accumulation. Lipofuscin accumulation reduces the efficacy of autophagy enzymes, resulting in a significant decrease in autophagy activity [17,18,19,20,21,22].

### 1.4. Impaired Regeneration of the Aged Liver is Related to Impaired Autophagy

Liver regeneration requires abundant energy and cellular substances for DNA replication and cell division [23]. Autophagy can effectively provide the needed substances during the regenerative period and remove dysfunctional organelles or aggregated proteins [11,13]. Both contribute to the coordinated proliferation of hepatocytes during the regenerative process.

Recent experiments have shown that there is a close link between autophagy and liver regeneration, but its role is discussed controversially [5,13,24,25,26,27,28,29]. Most studies have shown that a moderate induction of autophagy can promote liver regeneration, but some studies have reached the opposite conclusion. However, little is known about the impact of old age on the inter-related processes of autophagy and regeneration,

In this review, we want to

(1) clarify the relationship of intermingled molecular pathways of liver regeneration and autophagy in the aged liver and

(2) identify potential pharmacological strategies to induce autophagy and thereby restore the age-related impaired liver regeneration.

## 2. Liver Regeneration

### 2.1. The Powerful Regenerative Capacity of the Liver Is the Pathophysiological Basis for Successful Partial Hepatectomy

The liver consists of parenchymal cells (hepatocytes) and non-parenchymal cells (Kupffer cells, endothelial cells, epithelial cells, stellate cells and lymphocytes). Under normal physiological conditions, most of the hepatocytes are quiescent. The liver has the unique ability to switch from a quiescent to a proliferative state in response to a loss of liver cells due to surgery or chemical injury.

For example, hepatocytes enter into the cell cycle and start mitosis after partial hepatectomy (PH) (of various extents), portal vein ligation (PVL), acute toxic insult, viral infection and other types of stimuli.

Partial hepatectomy (PH) in rats or mice is a widely used model for studying liver regeneration. After 2/3PH, the residual hepatic tissue almost completely restores the original mass and function in about one week, demonstrating the amazing regenerative ability and compensatory functional capacity [30,31,32].

### 2.2. Under Most Circumstances Regeneration of the Liver Is Achieved by the Division of the Remaining Mature Hepatocytes

**Figure 1:**
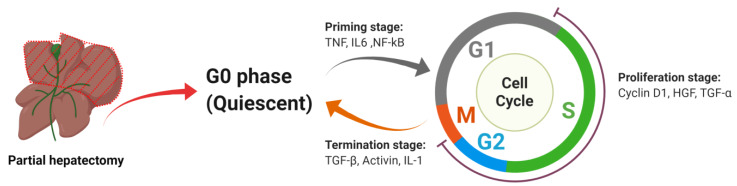
Overview of liver regeneration stages. After partial hepatectomy, every stage is governed by specific transcription factors and cytokines. Under their tight regulation, hepatocytes undergo the process from initiation to termination of proliferation.

After loss of a substantial amount of liver mass, the remnant mature hepatocytes start to divide rapidly to regenerate the organ to approximately full size. Liver regeneration via proliferation of hepatocytes is a highly complex process, consisting of three stages: the priming stage, the proliferation stage and the termination stage. Each of the stages is controlled by specific transcription factors and cytokines resulting in a highly regulated process (Figure 1), which leads to restoration of the liver mass within days.

#### 2.2.1. Priming Stage

Quiescent hepatocytes switch from G0 to G1 of the cell cycle within 4 h after partial hepatectomy both in rats and mice [33]. The cytokines TNF, IL6 and transcription factor NF-kB are three key regulators in this stage.

##### TNF

Tumor Necrosis Factor (TNF) is a multi-function cytokine. It is mainly produced by macrophages. The main effect of TNF is to regulate immune cells. It is involved in a variety of cellular processes, such as inflammatory responses, apoptosis, cell differentiation and proliferation.

TNF is secreted in the liver within 1 h in response to partial hepatectomy [34]. It binds to Tumor Necrosis Factor Receptor 1 (TNFR-1) in non-parenchymal cells in the liver and activates Nuclear Factor Kappa B (NF-kB). The activated NF-kB shifts to the nucleus to induce IL-6 expression, which in turn, activates Signal Transducer and Activator of Transcription 3 (STAT-3), Extracellular Signal-Regulated protein Kinases 1 and 2 (ERK1/2) pathways. Activated STAT3 enters the nucleus to initiate transcription of hepatocyte growth response genes. ERK1/2 can promote hepatocyte proliferation and DNA replication [35,36,37,38,39].

Talarmin [40] observed in a rat experiment that ERK activation occurred in the G1 phase in hepatocytes of liver-resected rats undergoing regeneration, but ERK activation was not found in livers from sham-operated rats. Yamada [41] established a *TNFR-1* knockout model and observed severe impairment of liver regeneration in the mice, which confirms the importance of TNF signaling in liver regeneration.

##### IL6

Interleukin-6 (IL-6) is a pleiotropic cytokine that is also involved in several pathophysiological activities, such as immune responses, apoptosis and cell proliferation. It is considered as being an effective hepatocyte mitogen [42].

IL-6 first binds to the IL-6 receptor (IL-6R) to form a complex. Due to a change of configuration, it can bind to Glycoprotein 130 (gp130) to form a high-affinity complex with signal transduction properties [43,44,45,46]. After hepatectomy, Lipopolysaccharide (LPS) and other gut-derived factors activate Kupffer cells, resulting in a TNF-α-dependent secretion of IL-6 [36]. Subsequently, the elevation of IL-6 activates the transcription factors STAT3 and CCAAT/enhancer-binding protein beta (C/EBPβ)/nuclear factor-interleukin 6, leading to increased transcription of their target genes.

Lack of IL6 expression leads to an impairment of liver regeneration as observed in *IL-6*^−/−^ mice. Liver morphology was characterized by ballooning degeneration and necrosis of hepatocytes. Furthermore, decreased DNA synthesis in hepatocytes and lack of STAT3 expression was observed in this study. Interestingly, this defect was limited to hepatocytes because the DNA synthesis seemed normal in *IL-6*^−/−^ non-parenchymal cells [47]. Thus, the transcription factors and cytokines mentioned above might contribute to the transition of hepatocytes from G0 to G1 phase after liver resection [48,49,50,51,52,53,54].

##### NF-kB

Nuclear factor kappa B (NF-kB) is a transcription factor that is involved in the inflammatory response, apoptosis, cell survival and proliferation. It is a heterodimer, which consists of two components—RelA and p50. However, the heterodimer is inactive when IκB binds to the RelA subunit. Activation of NF-kB requires the elimination of IκB from the heterodimer. The IκB kinase (IKK) can phosphorylate IκB and trigger its degradation. Once it is degraded, the activated NF-kB heterodimer migrates to the nucleus, where it binds with the target gene to promote gene transcription [33,55,56].

NF-kB is activated within 30 min after partial hepatectomy [55,56,57]. Iimuro [58] observed that blocking NF-kB by a NF-kB inhibitor (Ad5IkB) during liver regeneration caused a large number of hepatocytes to undergo apoptosis, indicating that NF-kB plays a positive role in preventing apoptosis during the course of liver regeneration.

#### 2.2.2. Proliferation Stage

Hepatocytes cross the restriction point of the G1 phase stimulated by mitogens. Then, they enter the synthesis and mitosis phase. HGF, TGF-α and Cyclin D1 play an important role in this stage.

##### Cyclin D1

Cyclin D1 is a key mediator for hepatocyte proliferation and is also one of the major markers of the hepatocyte entry into the cell cycle. Cyclin D1 expression is increased after 70% PH in animals. The Cyclin D1/Cyclin-dependent kinase 4 (CDK4) complex facilitates cells crossing the G1 restriction point and entering the G1/S transition phase in the cell cycle by phosphorylating Retinoblastoma (Rb) and E2F [33,59]. In Jeffrey’s observation [60], he found the expression of Cyclin D1 promoted hepatocytes to cross the G1 restriction point under the stimulation of epidermal growth factor (EGF). Furthermore, overexpression of Cyclin D1 in primary hepatocytes elevated the level of DNA replication even in the absence of growth factors.

##### HGF

Hepatocyte growth factor (HGF) is a potent growth factor mainly derived from activated hepatic stellate cells (HSC). HGF is a hepatocyte-specific mitogen that promotes hepatocyte proliferation and DNA synthesis [61,62,63]. Lindroos [64] found that the plasma level of HGF increased sharply after partial hepatectomy in rats. HGF was about 17-fold higher in liver-resected rats compared to normal animals, and HGF elevation did persist for more than 24 h.

##### TGF-α

Transforming growth factor alpha (TGF-α) is a member of the EGF ligand family and acts also as a mitogen for hepatocytes. It promotes hepatocyte proliferation by binding to EGF receptor (EGFR). TGF-α can significantly promote DNA synthesis in hepatocytes [65,66,67,68]. Mead [68] indicated that the TGF-α mRNA increased by about 9-fold in regenerating rat livers compared to normal livers during the peak of DNA synthesis. Treating primary hepatocytes with TGF-α resulted in a 13-fold increase of DNA synthesis.

#### 2.2.3. Termination Stage

Once the volume and weight of the liver return to the preoperative level, the proliferation ceases. Hepatocytes terminate proliferation under the control of TGF-β1, Activin and IL-1.

##### TGF-β

Transforming Growth Factor beta (TGF-β) is mainly secreted by hepatic stellate cells in the liver. It can inhibit the synthesis of hepatocyte DNA. It can also induce apoptosis and contribute to size regulation during regeneration [69,70,71].

TGF-β family messenger RNA and protein levels are upregulated in quiescent hepatocytes where most cells are in the G0 phase. TGF-β is inhibited in the early stage of regeneration by up-regulation of TGF-β inhibitory proteins Ski-related novel protein N (SnoN) and Sloan-Kettering Institute (Ski). Down-regulation of the TGF-β receptors prompt hepatocytes to transition from G1 to S phase. However, it is restored during termination stage of liver regeneration, driving the hepatocytes into a quiescent state [36,72,73].

##### Activin

Activin is a member of the TGF-β family. As well as most other members of the family, it is a dimeric protein. It is involved in regulating cell differentiation and homeostasis [74,75].

Activin is a DNA synthesis inhibitor of hepatocytes [70,76]. Takamura [77] observed that Activin was reduced in the early stage of liver regeneration after partial hepatectomy, but it increased significantly at 120 h. Multiple studies showed it can induce hepatocyte growth arrest and apoptosis [76,78,79,80].

##### IL-1α/β

The IL-1 family contains 11 cytokines; the most studied cytokines are IL-1α and IL-1β. IL-1α/β are pro-inflammatory cytokines and they play an important role in the inflammatory response, cell proliferation and differentiation [81]. Numerous studies showed that IL-1α/β are negative regulators during liver regeneration.

IL-1α/β are secreted by Kupffer cells and other non-parenchymal cells in the regenerating liver [82]. Both cytokines inhibit hepatocyte proliferation induced by liver resection. Boulton [83] found that the expression of IL-1α mRNA was down-regulated in the early stage of liver regeneration at about 10 h postoperatively. However, IL-1α mRNA was up-regulated at 24 and 48 h when the proliferation intensity decreased in rat livers after liver resection. The IL-1β expression pattern was similar to IL-1α. Similarly, Nakamura [84] observed in-vitro that IL-1β significantly impaired DNA synthesis in primary rat hepatocytes stimulated by insulin and EGF. The above results show that IL-1α/β is involved in the terminating excessive proliferation of hepatocytes.

### 2.3. The Effects of Aging on Liver Morphology, Metabolism and Regeneration

Aging causes morphological and physiological changes in the liver, such as a decrease in hepatic volume and perfusion [4]. It leads to an increase in the size of hepatocytes, a decrease in the number of mitochondria and an increase in the number of binucleated cells [10,85]. However, the aging liver can maintain its metabolic function relatively well. Structural analysis of the liver showed that mitochondrial integrity did not undergo an obvious age-related change. No significant change in liver enzyme activity was observed [3,86].

In contrast, aging significantly impedes liver regeneration [10]. After partial hepatectomy, 90%–100% of the hepatocytes in the young liver enter the S phase, while only about 30% of the hepatocytes in the aged liver enter the S phase [87].

### 2.4. Liver Progenitor Cells may Enhance Liver Regeneration under Certain Circumstances

In addition to the division of mature hepatocytes, the first line of defense for liver regeneration, liver progenitor cells (LPCs) are also shown to participate in the course of animal liver regeneration [88,89]. This is called the second line of defense for liver regeneration (Table 1).

LPCs, also described by some authors as oval cells, are bipotent progenitor cells residing in the canal of Hering at the transition between bile canaliculi and bile ductuli. They can differentiate into hepatocytes and cholangiocytes [29,90].

This is described in two conditions: (1) when the liver is severely damaged, e.g., by the administration of 2-acetylaminofluorene administration before PH; (2) when the liver is subjected to chronic injury, e.g., by administration of a Choline-Deficient, Ethionine-supplemented (CDE) diet. In both cases, the remaining hepatocytes cannot meet the needs of liver regeneration. As a consequence, LPCs are activated and contribute to liver regeneration by renewing and differentiating into hepatocytes and cholangiocytes [91,92].

However, the contribution of LPCs for liver regeneration was questioned by some authors [93,94]. For example, using the mouse model of chronic liver injury induced by CDE diet, Schaub [93] could not trace hepatocytes which were clearly derived from liver stem cells (LSCs). One possibility they considered was that their model of liver injury was insufficient for LSCs activation.

### 2.5. The Effects of Aging on Liver Progenitor Cells

The ability of LPCs to respond to hepatic injury declines with age. Cheng observed that [89] LPCs in young mice can be activated and proliferate properly after CDE diet-induced hepatic injury. However, LPCs in old mice failed to respond to the injury, resulting in impaired liver regeneration. Further research showed that hepatic stellate cells in old mice secreted more Chemokine (C-X-C motif) ligand 7 (CXCL7) compared with young mice, which induced neutrophil infiltration in the liver. Neutrophil infiltration produced excessive reactive oxygen species (ROS), thus impairing the activation and proliferation of LPCs to limit the regeneration of liver.

Autophagy plays a vital role in maintaining the function of LPCs as indicated by three different observations. First, Cheng [29] found LPCs had higher autophagy activity compared with differentiated hepatocytes. After inhibiting autophagy by knocking down the *Atg5* or *Beclin1* genes, self-renewal, proliferation and hepatic-differentiation capacity of LPCs were significantly impaired. Second, inhibition of autophagy makes LPCs more vulnerable to senescence induced by Etoposide stimulation. In contrast, overexpression of *Beclin 1* in *Beclin1* knockdown (KD)-LPCs restored their hepatic-differentiation capacity and increased its resistance to Etoposide-induced senescence. Third, Ma [102] also observed a similar result that inhibiting the expression of *Atg5* seriously hampered hepatic differentiation of LPCs. These results reflect the important role of autophagy in hepatic differentiation of LPCs.

## 3. Autophagy

After partial hepatectomy, the loss of liver mass triggers autophagy to provide the necessary energy, thereby creating an optimal environment for regeneration. However, hepatic autophagy levels gradually decline with age [5,14,15,16]. Induction of autophagy may be an effective way to promote the recovery of liver mass and physiological function.

### 3.1. Autophagy Is an Essential Mechanism for Eukaryotes to Recycle Intracellular Components

Three forms of autophagy are currently known: macroautophagy, microautophagy and chaperone-mediated autophagy. Here, we mainly discuss macroautophagy, which is closely related to liver regeneration.

**Figure 2:**
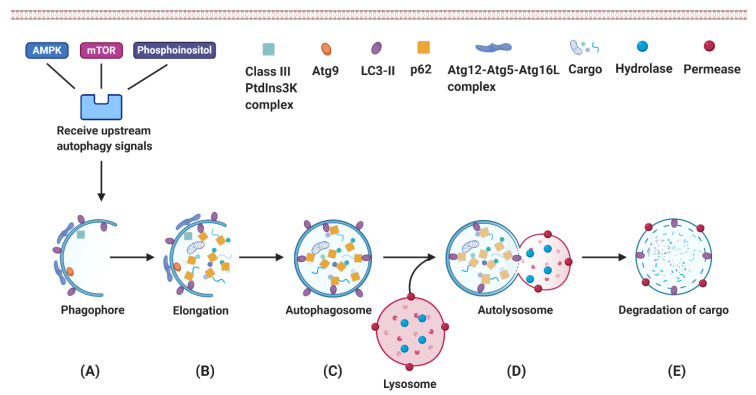
The dynamic process of autophagy. Macroautophagy mainly includes the following steps: (**A**) Phagophore formation. Activation of the Class III PtdIns3K complex produces PI3P, which facilitates the nucleation of phagophores and regulates the degradation of autophagy cargo [106]. (**B**) Phagophore elongation and capture of degradation targets. The function of the transmembrane protein Atg9 is to deliver the membrane from donor organelles to the expanding phagophore [107]. The LC3-II and Atg12–Atg5–Atg16L complex promotes phagophore elongation [106]. (**C**) Autophagosome formation. p62 interacts with the autophagy cargo and delivers it to the autophagosome. (**D**) Fusion of the autophagosome with lysosome; (**E**) Degradation of the cargo.

Macroautophagy is the most common type of autophagy (Figure 2). Activation of the autophagy signaling cascade leads to the formation of phagophore as the very first step. The phagophores derive from the omegasome, a cell compartment rich in phosphatidylinositol-3-phosphate (PI3P) on the endoplasmic reticulum. As a next step, phagophores continue to extend and capture autophagy cargo in the cytoplasm. They eventually form the bi-membrane layered autophagosome. Autophagosomes fuse with lysosomes to deliver the autophagy cargo into the lysosome. The cargo is digested by lysosomal hydrolases into amino acids, fatty acids and other basal components, which are then released by membrane permease for further reuse [103,104,105].

Autophagy is divided into basal autophagy and induced autophagy. Basal low-level autophagy occurs constantly in most cells since it is vital for intracellular homeostasis and cell self-renewal. In contrast, induced high-level of autophagy is mainly observed in cells under stress, since it represents a protective response to compensate injury [105,108,109]. Under stress conditions, moderate-level autophagy contributes to cell survival but excessive autophagy may cause cell death [110,111].

### 3.2. Autophagy Plays an Essential Role in Hepatic Physiological Processes

Autophagy is one of the pro-survival processes allowing hepatocytes to resist cellular stressors such as anoxia and nutrient starvation [112]. Autophagy protects hepatocytes from damage and cell death by clearing damaged organelles and misfolded proteins generated in hepatic disease [113].

The liver is the largest metabolic organ in the human body, and autophagy plays a vital role in metabolic activities. Autophagy is involved in glucose, lipid and protein metabolism of the liver [114]. Liver autophagy converts amino acids into glucose through gluconeogenesis, a fundamental process for maintaining blood glucose level [115].

The process whereby autophagy degrades lipid droplets is termed Lipophagy. The liver is the body’s second-largest “storeroom” of lipids. Lipophagy can degrade lipid droplets into free fatty acids (FFAs), and these FFAs are oxidized in mitochondria to produce Adenosine Triphosphate (ATP) [114,116]. In case of metabolic needs due to a lack of nutrients such as starvation or due to an increased demand as in regeneration, the autophagic cargo is degraded, resulting in an enhanced ATP production as needed for the given processes.

### 3.3. Autophagy Provides Energy as Needed for Liver Regeneration

It is well known that liver regeneration requires abundant ATP to support the energy-consuming process of cell division and growth [23]. Hepatocytes are rich in mitochondria to meet the energy needs such as their multitudinous metabolic activities and liver regeneration. However, liver resection can cause massive mitochondrial damage and reduce ATP production in hepatocytes, leading to a significantly reduced availability of ATP. Toshima [13] observed a substantial decline of ATP reserves as early as 6 h after mouse liver resection.

In the initial stage of liver regeneration, autophagy, especially mitochondrial selective autophagy (Mitophagy), is essential for maintaining healthy mitochondria that can produce ATP [105,117]. Mitochondria are highly dynamic organelles that continuously fuse, divide (termed mitochondrial dynamics) and form a network structure within the cells [118]. Mitochondrial dynamics manage mitochondrial morphology, distribution and quality [119,120,121]. During this process, mitophagy selectively degrades damaged or dysfunctional mitochondria to promote mitochondrial regeneration and maintain ATP synthesis [122,123,124]. This process provides the energy needed for liver regeneration.

### 3.4. Age-Related Alterations Impair Autophagy Activity

#### 3.4.1. Age-Related Decline of AMPK Activation Impairs Autophagosome Formation

AMP-activated protein kinase (AMPK) is the main cellular energy sensor. It is activated when cells are in a low energy state indicated by an increased AMP/ATP ratio [125]. Activation of AMPK can effectively induce the onset of autophagy. However, aging causes the decrease of the activation capacity of AMPK, which impairs autophagosome formation, disrupts the maintenance of homeostasis in cells and further accelerates the aging process [20,21,22].

#### 3.4.2. Age-Related Lipofuscin Accumulation in Lysosomes Reduces the Efficiency of the Degradation Process

Lipofuscin is an intracellular cross-linked polymer, consisting of protein residues. The formation of lipofuscin is mainly due to the iron-catalyzed oxidative damage of macromolecules. It is generally accumulating in lysosomes of most cells (including liver cells) with age [126,127].

The lysosome is one of the most important subcellular components in the process of autophagy. Lysosomes maintain an acidic microenvironment to ensure optimal operating conditions for lysosomal enzymes. However, some of the autophagic cargo remains hard to digest. For instance, some damaged and denatured proteins after being exposed to oxidative stress are not degraded, which leads to the formation of lipofuscin.

When lipofuscin-loaded lysosomes accumulate in senescent cells, most lysosomal enzymes are directed from the Golgi-apparatus to the lipofuscin-loaded lysosomes. However, the lysosomal enzymes cannot degrade lipofuscin. In consequence, delivery of enzymes to the lipofuscin-loaded lysosomes is not effective in terms of recycling the damaged proteins. This imbalanced distribution decreases the availability of autophagic enzymes in healthy non-lipofuscin loaded lysosomes [17,18,19], altogether resulting in a further impairment of the autophagy process.

Moreover, lipofuscin accumulation leads to a reduced turnover of damaged mitochondria, which results in an increase in reactive oxygen species. In turn, the oxidative stress further impedes autophagy via the additional impairment of lysosomal function [128,129,130,131,132].

In conclusion, the accumulation of lipofuscin leads to a decline in the lysosomal capability to break down the intracellular macromolecules, resulting in substantial impairment of autophagy.

### 3.5. Autophagy Can Be Modulated by Interfering with Key Signal Transduction Pathways

**Figure 3:**
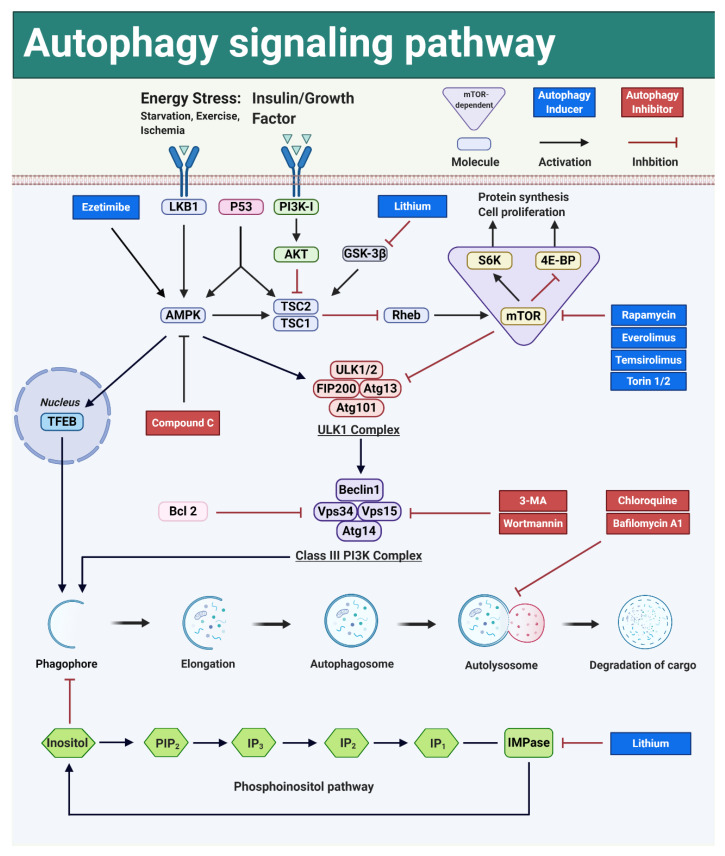
Signaling pathways and modulators involved in the regulation of macroautophagy. mTOR is a key regulator for both autophagy and cell proliferation. Autophagy can be activated in the mTOR-dependent and the mTOR-independent signaling pathways. Dark blue square: autophagy inducer; Dark red square: autophagy inhibitor.

Autophagy is regulated via mammalian target of rapamycin (mTOR)-dependent and mTOR-independent signal transduction pathways (Figure 3). mTOR is a kinase centrally involved in the molecular control mechanism governing cell proliferation, immune responses and metabolic processes, but is also of utmost importance in autophagy [133].

The autophagy pathways are named based on the key molecules involved. Currently, three mTOR-dependent pathways are considered to be important: the PI3K-AKT-mTOR pathway, the LKB1-AMPK-mTOR pathway and the P53-AMPK-mTOR pathway. Furthermore, also three, but less well-explored mTOR-independent pathways are of importance: the AMPK-ULK1 pathway, the AMPK-TFEB pathway and the Phosphoinositol pathway.

With the deepening of autophagy research, an increasing number of autophagy inducers are available (Table 2). According to their mechanism of action on autophagy, they are divided into mTOR-dependent and mTOR-independent autophagy inducers [134,135].

#### 3.5.1. Autophagy Can Be Activated by Interfering with the mTOR-Dependent Signal Transduction Pathways

In the following paragraphs, we are first introducing the pathways, followed by explaining the mechanism of action of key drugs and are then discussing their potential to modulate liver regeneration.

##### PI3K-AKT-mTOR

The best described pathway is the PI3K-AKT-mTOR pathway. The Phosphatidylinositol 3-Kinase (PI3K) protein family is involved in the regulation of autophagy, but also in various other cellular functions such as cell proliferation, differentiation and apoptosis.

Activation of PI3K via phosphorylation leads to the generation of a second messenger called phosphatidylinositol-3,4,5-triphosphate (PIP3), which binds to signaling kinases AKT and PDK1 (phosphoinositide-dependent kinase-1). AKT is phosphorylated by PDK1. Activated AKT can directly phosphorylate mTOR and thereby activate mTOR to inhibit autophagy. Moreover, AKT enriches the GTP-binding protein-Ras homolog enriched in brain (Rheb) by phosphorylating Tuberous sclerosis complex 1/2 (TSC1/2), then up-regulating mTOR to inhibit autophagy [141,142,143].

mTOR is capable of interacting with multiple binding proteins to form two complexes with different structures and functions—named mTOR complex 1 (mTORC1) and mTOR complex 2 (mTORC2).

mTORC1 regulates several pathways that together determine the size of the cell. In mammals, it is primarily responsible for the regulation of cell proliferation and autophagy, but also for protein synthesis and ribosome biosynthesis.

mTORC1 is a negative regulator of autophagy. When nutrients such as amino acids are abundant, the activity of mTORC1 is elevated to down-regulate autophagy. In the absence of nutrients (especially glucose), AMPK is up-regulated, which causes mTOR to be down-regulated, thereby inducing autophagy. mTOR acts on two important downstream proteins: S6K1 (p70S6 kinase 1) and eIF4E binding protein (4E-BP). Their main role is to regulate cell growth, proliferation and protein synthesis [141,144,145].

mTORC2 is relatively insensitive to Rapamycin, it mainly regulates the actin cytoskeleton to organize the cell shape and modulates cell metabolism [146].

##### LKB1-AMPK-mTOR

The LKB1-AMPK-mTOR pathway is involved in the regulation of autophagy, metabolism and cell growth. The liver kinase B1 (LKB1) is a tumor suppressor that acts on the upstream kinase of AMPK. LKB1 can phosphorylate and activate AMPK, which is a key signaling molecule in autophagy as well as in glucose and lipid metabolism [147,148].

AMPK is considered to be a major energy-sensing kinase that activates a variety of catabolic processes. mTORC1 senses the energy state of cells through AMPK, which is activated in response to low intracellular energy levels. Activated AMPK enhances autophagy to promote the production of ATP [146].

Previously, AMPK was thought to regulate mammalian autophagy through the mTOR pathway. AMPK phosphorylation of TSC2 indirectly leads to inhibition of mTOR by inactivating Rheb. Inhibition of mTOR restores the activity of UNC-51-like kinase 1 (ULK-1)—a key autophagy promoter—thereby activating autophagy [149,150,151,152,153,154].

##### p53-AMPK-mTOR

The p53-AMPK-mTOR pathway is mainly involved in the regulation of autophagy and cell growth. p53—a human tumor suppressor protein—activates AMPK, leading to the inhibition of mTOR [155].

Evidence is accumulating that p53 may regulate autophagy in two-ways according to the subcellular localization. In the nucleus, active p53 tetramers bind to a promoter region of multiple genes encoding for autophagy regulators such as AMPK, TSC2 and death-associated protein kinase 1 (DAPK-1). In contrast, cytoplasmic p53 inhibits autophagy, but its related mechanism remains unclear [156].

Furthermore, p53 can indirectly affect cell growth and proliferation by activating cell cycle regulators. For example, p53 activates its downstream factor p21, which is a Cyclin-dependent kinase (CDK) inhibitor that causes cell cycle arrest [156].

##### mTOR-Dependent Autophagy Induction and Liver Regeneration

The mTOR-dependent signaling pathways are not only crucial for autophagy, but are also of utmost importance for cell proliferation.

Rapamycin, a macrolide immunosuppressant, is a typical mTOR inhibitor. It has been used to combat fungal infections and to suppress immune functions in organ transplant recipients [157]. Nowadays, it has attracted much attention as an mTOR-dependent autophagy inducer. It can induce autophagy in yeast even under nutrient-rich conditions [158]. Rapamycin activates autophagy by inhibiting the activity of mTOR. It inhibits mTORC1 via forming a compound with FK506 binding protein 12. The complex acts on downstream proteins to inhibit protein synthesis and leads to cell cycle arrest by preventing the transition from G1 to S phase [146,159]. This anti-proliferative effect has been confirmed in several studies of liver regeneration after partial hepatectomy [160,161]. Similar effects were observed when using other mTOR-dependent inducers such as Temsirolimus [27].

In conclusion, mTOR inhibitors can induce autophagy via the mTOR-dependent pathway. However, this may severely impede cell proliferation. Therefore, mTOR-dependent autophagy inducers are not suitable for promoting liver regeneration.

#### 3.5.2. mTOR-Independent Autophagy Pathways Are Not Interrelated with Cell Proliferation Pathways

Inducing autophagy without inhibiting cell proliferation as needed for liver regeneration is the better alternative. Here, modulating the mTOR-independent pathways may have the potential for promoting liver regeneration. As in the previous chapter, we are first introducing the pathways, then present potential mTOR-independent autophagy inducers and discuss their potential to augment regeneration.

##### AMPK-ULK1

Recent studies have shown that there is another pathway downstream of AMPK influencing autophagy, the AMPK-ULK1 pathway. AMPK directly phosphorylates ULK1, which in turn, initiates the formation of the autophagosomes [162,163].

Several observations support this mechanism:

First, Guha [164] elucidated this pathway when studying the impact of IPMK on autophagy. Inositol Polyphosphate Multikinase (IPMK) is a potent enzyme that catalyzes the production of inositol polyphosphate and phosphatidylinositol. *IPMK* deletion in mouse embryonic fibroblasts (MEFs) abrogated the AMPK-related ULK phosphorylation, thereby decreasing the autophagy level. The deletion of *IMPK* down-regulated autophagy and suppressed hepatocyte proliferation by approximately 50% in a mouse liver regeneration experiment using the Carbon Tetrachloride (CCL4) model.

Second, Liu further clarified this pathway when investigating the impact of young plasma on age-impaired autophagy. He [5] first observed in aged rats that treatment with young plasma promoted impaired liver regeneration through the restoration of age-impaired autophagy. In a subsequent study [14], he revealed in a rat model of liver ischemia-reperfusion injury (IRI) that young plasma restored age-impaired autophagy at least partially through the AMPK-ULK1 signaling pathway. In old rats (22 months) treated with young plasma, AMPK phosphorylation was increased, leading to ULK1 activation. Furthermore, in the corresponding cell culture experiment, inhibition of AMPK activity with an AMPK inhibitor (Compound C) prevented the activation of ULK1 and thereby prevented the initiation of autophagy induced by young serum. In other words, treatment with the AMPK inhibitor abolished the protective effect of young serum on hypoxia/re-oxygenation injury in aged hepatocytes, supporting the key role of autophagy for this effect [14]. Taken together, his observations suggest that induction of autophagy via the AMPK-ULK1 pathway may also have the potential for enhancing liver regeneration.

##### AMPK-TFEB

The transcription factor EB (TFEB) is one of the critical components in lysosomal biogenesis and autophagy. Furthermore, TFEB regulates the expression of genes involved in different phases of the autophagy process [165,166].

Kim [167] observed that TFEB-induced autophagy was dependent on the AMPK pathway. After inhibiting AMPK activity via an *AMPK*-specific small interfering RNA (siRNA), autophagy was no longer activated through the TFEB pathway.

The activity of the TFEB is mainly affected by its subcellular localization. In the cytoplasm, phosphorylated TFEB suppresses the transcriptional activation of its target genes (e.g., *BECN1, UVRAG* and *RAB7*). Once TFEB is transferred to the nucleus, as in starvation or in lysosomal storage disease (e.g., Gaucher disease), it is dephosphorylated and binds to the target genes [165,166], which promotes autophagosome formation and its fusion with lysosomes.

##### Phosphoinositol Pathway

Autophagy can also be activated via decreasing Inositol or Inositol 1,4,5-trisphosphate (IP_3_) levels [168]. Inositol 1,4,5-trisphosphate (IP_3_) negatively regulates autophagy, but the specific mechanism is still unclear.

Two potential mechanisms, both proposed by the group of Kania, are currently discussed. On the one hand, Kania [169] proposed that the endoplasmic reticulum (ER), the organelle storing most of the intracellular Ca^2+^, can release Ca^2+^ after IP_3_ binds to membraneous IP_3_ receptor on its surface. This step is considered to be the prerequisite for maintaining the mitochondrial energy state. Providing Ca^2+^ to mitochondria promotes the production of Nicotinamide adenine dinucleotide (NADH) and ATP. In contrast, inhibition of IP_3_ or IP_3_ receptor through pharmacological modulation or using gene knockout resulted in a reduced ATP production. Decreased energy levels activate AMPK and induce autophagy via an mTOR-independent mechanism to maintain energy homeostasis [170].

On the other hand, Kania also proposed, that autophagy can be induced by inhibition of IP_3_ receptor. Beclin 1 promotes autophagy by forming a class III PI3K complex with Atg14, Vps34, Vps15 [138,169], this complex is essential for autophagosome formation. IP_3_ receptor can bind to Beclin 1 since IP_3_ receptor may become a target of Beclin 1 recruitment, which diminishes the probability of Beclin 1 for promoting autophagy [171].

##### mTOR-Independent Autophagy Inducers and Liver Regeneration

Currently a number of mTOR-independent autophagy inducers are available. Based on this pathway analysis, mTOR-independent autophagy inducers should not interfere with liver regeneration. For two of those drugs, recent studies confirmed that inducing autophagy via the mTOR-independent pathways indeed did promote liver regeneration in liver-resection models.

Carbamazepine is a common anticonvulsant, which is clinically often used to treat epilepsy and neuropathic pain. Carbamazepine can induce autophagy through depletion of cellular inositol and AMPK activation. The depletion of cytosolic inositol leads to a reduction in basal IP_3_, which reduces ATP production by impeding mitochondrial Ca^2+^ entry. Then, the decreased ATP concentration can activate the AMPK-ULK1 pathway to induce autophagy [172,173].

Kawaguchi [27] recently presented the impact of Carbamazepine-treatment on liver regeneration after partial hepatectomy in mice. Treatment with Carbamazepine significantly promoted hepatocyte proliferation by activating mTOR and its downstream factors S6K. Compared with the animals in the control group, the Liver to Body Weight Ratio (LBWR), Proliferating Cell Nuclear Antigen (PCNA), Ki-67 and 5-Bromo-2′-Deoxyuridine (BrdU) index of Carbamazepine-treatment animals increased significantly on the post-operation day 2 (POD2).

Amiodarone is an antiarrhythmic drug. It is commonly used to treat tachycardia and ventricular fibrillation. It is currently gaining attention as an autophagy inducer. Amiodarone-treatment blocks extracellular Ca^2+^ afflux via inhibiting L-type Ca^2+^ channels on the plasma membrane, thereby reducing the intracellular Ca^2+^ level [140]. Decreasing the intracellular Ca^2+^ level can activate autophagy [174,175].

Lin [26] observed in a mouse model of PH that Amiodarone can substantially increase autophagy flux via the mTOR-independent pathway and enhance liver regeneration. The authors observed significantly higher LC3-II levels in animals of the amiodarone-treated group compared to the control group, but significantly lower p62 levels. The LBWR and Ki-67 index increased substantially in these mice, indicating an enhanced hepatic proliferative response. In contrast, inhibition of autophagy by *Atg7* knockdown or Chloroquine pretreatment caused a significant impairment in liver regeneration.

However, for a number of other mTOR-independent autophagy inducers, the impact on liver regeneration was not yet demonstrated. Based on their mode of action, peri-operative treatment with these drugs should also promote liver regeneration.

Ezetimibe is a Niemann-Pick C1-Like 1 (NPC1L1) inhibitor, which is currently used for the treatment of hypercholesterolemia. It has been proven to improve hepatic steatosis and protect hepatocytes from excessive apoptosis by activating autophagy [167,176,177].

Kim [167] observed that Ezetimibe improved lipid metabolism and reduced hepatocyte apoptosis by inducing autophagy. Ezetimibe promoted autophagy via AMPK activation and TFEB nuclear translocation. TSC2 is a negative modulator of mTOR. Knocking out *TSC2* can activate mTOR. It is noteworthy that Ezetimibe still effectively induced autophagy in the *TSC2*^−/−^ MEFs. Treatment with Ezetimibe upregulated *p*-AMPK in the cells. Further experiments revealed that under the mTOR-activated condition, Ezetimibe-treatment promoted TFEB nuclear translocation via inhibition of Mitogen-Activated Protein Kinase (MAPK)/Extracellular Signal-Regulated Kinase (ERK) signaling in primary hepatocytes.

Moreover, Yu [176] observed in a rat model of middle cerebral artery occlusion that Ezetimibe promoted the phosphorylation of AMPK, which induced autophagy through directly activating ULK1. Consequently, enhanced autophagy alleviated neuronal apoptosis. Pretreatment of the rats with an AMPK inhibitor (Dorsomorphin) inhibited autophagy and abolished the favorable effects of Ezetimibe. Given that Ezetimibe can induce autophagy and effectively promote hepatocyte survival, it deserves further study regarding its value in liver regeneration.

Lithium is widely used in the treatment of mental disorders such as manic depression. Lithium can activate autophagy without reducing the activity of mTOR and its downstream factors *p*-S6K1 and *p*-4E-BP1. It activates autophagy by the inhibition of inositol monophosphatase, causing the depletion of inositol and reduces IP_3_ levels [168].

Moreover, Lithium inhibits Glycogen Synthase Kinase-3 Beta (GSK-3β) signaling, which activates mTOR by inhibiting TSC2. Nonetheless, Lithium can effectively induce autophagy via the Phosphoinositol pathway in mTOR-activated condition, since these pathways are independent from each other [178]. In conclusion, it is also of high interest to explore the potential of Lithium for promoting liver regeneration.

## 4. Interactions between Autophagy, Liver Regeneration and Aging

In summary, aging leads to the accumulation of lipofuscin in the lysosome, which impairs the efficiency of autophagic enzymes [17,18,19]. Moreover, aging causes a significant decrease in the number of autophagosomes, which may be related to the decline of activation capacity of AMPK. It further reduces autophagy activity [20,21,22].

As said before, liver resection not only triggers liver regeneration, but also induces autophagy of hepatocytes. Autophagy plays a crucial role in liver regeneration (Table 3) [5,13,26,28,29,164]. Liver regeneration requires abundant energy [23], which is among others generated by recycling intracellular macromolecules derived from organelles damaged during hepatectomy.

Autophagy activity in aged liver is significantly reduced compared to young liver [5,14,15]. Therefore, improving autophagy through pharmacological intervention seems to be an effective treatment to promote regeneration in senescent livers. The mTOR pathway is the most common autophagy-related pathway. However, the mTOR pathway is not only the key regulatory pathway for autophagy, but also the pathway that modulates cell proliferation. Inhibition of mTOR activity can induce autophagy, but inhibits cell proliferation at the same time. In the case of liver resection, inhibition of cell proliferation is detrimental, since it causes impairment of liver regeneration, as described in detail above.

**Figure 4:**
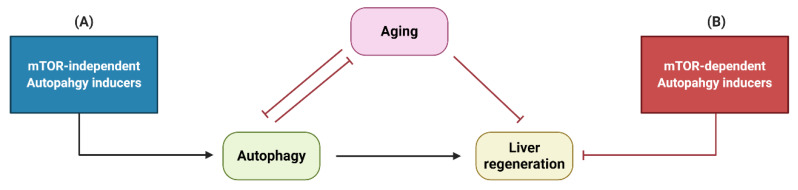
The relationship between autophagy, liver regeneration and aging. (**A**) mTOR-independent autophagy inducers can promote liver regeneration, while (**B**) mTOR-dependent autophagy inducers inhibit liver regeneration.

Therefore, modulation of autophagy via the mTOR-independent pathway is a better strategy (Figure 4). Strikingly different drugs such as Carbamazepine, Amiodarone, Ezetimibe and Lithium induce autophagy via these pathways. They are of documented or putative benefit for enhancing liver regeneration and should be explored in more depth. This is of special importance for the elderly population, where liver regeneration is already impaired, in part due to the age-dependent decrease of autophagic activity.

## 5. Conclusions

At present, many aging patients with malignant liver disease cannot be treated effectively because of the aging-related impairment of liver regeneration. Aging-related changes also lead to decreased autophagy activity, which is an important cause for insufficient liver regeneration. Age-specific strategies to promote liver regeneration for these patients at risk are needed.

Evidence is accumulating that the modulation of autophagy via pharmacological intervention is an effective approach to promote liver regeneration. This is of utmost benefit for aging patients with impaired autophagy. However, choosing the appropriate autophagy pathway to activate autophagy is crucial. Inducing autophagy by the mTOR-dependent pathway alone is detrimental to liver regeneration. In contrast, activation of autophagy via the mTOR-independent pathway does not affect cell proliferation. Therefore, mTOR-independent autophagy inducers such as Carbamazepine, Amiodarone, Ezetimibe and Lithium should be further explored to promote liver regeneration in the elderly patients.

## Figures and Tables

**Table 1:** Differentiation between first and second line of regeneration (based on an analysis of specific reviews). The regenerative capacity of the liver is built on two lines of defense. The 1st line of defense: division of remaining mature hepatocytes; 2nd line of defense: mainly through division and differentiation of liver stem/progenitor cells.

Modes of Liver Regeneration									
Year	Author	Conditions for 1st Line Regeneration			Conditions and Mechanism for 2nd Line Regeneration				Main Cell Type for 2nd Line of Regeneration (as Indicated by Author)
		Mild Liver Injury	CCL_4_^ΔΔ^	PH	Severe Liver Injury	Impaired Proliferative Capacity of Mature HCs ^🗶^	Chronic Liver Injury	Delayed Response to Hepatic Injury	
1996	Snorri [91]	**Y** **^Δ^**			**Y**				OCs *
2001	Nelson [95]			**Y**				**Y**	HSCs **/LPCs
2008	Viebahn [96]			**Y**			**Y**		LPCs
2013	Jan [97]	Not given				**Y**			LPCs
2013	Ioannis [98]	**Y**	**Y**	**Y**	**Y**		**Y**		PCs ***
2014	THAN [92]	**Y**		**Y**	**Y**	**Y**	**Y**		LPCs
2014	Itoh [90]			**Y**	**Y**	**Y**	**Y**		LPCs
2015	Jan [99]	**Y**				**Y**	**Y**		LPCs
2016	Minoru [100]	**Y**			**Y**		**Y**		LPCs
2017	Veronika [101]	Not given					**Y**		LPCs

^Δ^ The Y mark (Yes) indicates that the item is selected; ^ΔΔ^ CCL4: Carbon Tetrachloride. * OCs (Oval cells); ** HSCs (Hepatic stem cells); *** PCs (Progenitor cells); ^🗶^ HCs: Hepatocytes.

**Table 2:** Common autophagy inducer and their mechanism of action [136,137,138,139,140].

Commonly Used Autophagy Inducers		
Autophagy Inducers	Mode of ACTION	mTOR-Dependent
Rapamycin	mTOR inhibitor	Yes
Everolimus	mTOR inhibitor	Yes
Temsirolimus	mTOR inhibitor	Yes
Torins	mTOR inhibitor	Yes
Perifosine	AKT inhibitor	Yes
Ezetimibe	AMPK activator; MAPK/ERK inhibitor	No
Carbamazepine	Ins and IP_3_ * inhibitor	No
Sodium valproate	Ins and IP_3_ * inhibitor	No
Xestospongin B	IP_3_ receptor inhibitor	No
Xestospongin C	IP_3_ receptor inhibitor	No
Lithium chloride	IMPase ** inhibitor	No
Trehalose	Glucose transporter inhibitor; AMPK activator	No
Amiodarone	Calcium channel blocker	No

* Ins: Inositol; IP_3_: Inositol 1,4,5-trisphosphate. ** IMPase: Inositol monophosphatase.

**Table 3:** Controversy about role of autophagy in influencing regeneration.

(a). Scientific Evidence that Activating Autophagy Is Promoting Liver Regeneration							
Author Year	Research Model	Pathway	Autophagy Modulation	Parameters Indicating			
				Enhanced Autophagy	Enhanced Regeneration	Reduced Autophagy	Reduced Regeneration
Takeo [13] 2014	Mice	Not investigated	*Atg5*-KO			LC3-II: --- p62: +++	BrdU: ---
Lin [26] 2015	*C57BL/6* mice, Male	mTOR- independent	Amiodarone	LC3-II: +++ p62: ---	LBWR *: +++ Ki-67: +++		
			*Atg7*-KD			LC3-II: ---	LBWR *: --- Ki-67: ---
Cheng [29] 2015	Liver progenitor cells	Not investigated	Overexpression of *Beclin1*	LC3-II: +++	PAS ^×^: +++		
			*Beclin1*-KD; *Atg5*-KD			LC3-II: --- p62: +++	CCK-8: --- PAS: ---
Liu [5] 2018	*SD* rats, Male, 3m, 22m; Primary rat hepatocytes	Not investigated	Young plasma	LC3-II: +++ p62: ---	Ki-67: +++		
			3-Methyladenine; Wortmannin			LC3-II: ---	Ki-67: ---
Jia [28] 2019	*SD* rats, Male, 5w	Not investigated	70% Portal Vein Ligation	LC3-II: +++	Cyclin D1: +++		
Guha [164] 2019	Mice; MEFs; HEK293T cells.	IPMK-AMPK-ULK1; IPMK-AMPK-SIRT1	*IPMK*-KO			LC3-II: ---	Ki-67: --- Edu: ---
**(b). Scientific Evidence that Inducing Autophagy Is Inhibiting Liver Regeneration**							
**Author** **Year**	**Research Model**	**Pathway**	**Autophagy Modulation**	**Parameters Indicating**			
				**Enhanced Autophagy**	**Reduced Regeneration**	**Reduced Autophagy**	**Enhanced Regeneration**
Jiang [161] 2001	*Harlan SD* rats, Male	mTOR- dependent	Rapamycin	Not investigated	LWRR **: ---		
Palme [159] 2008	*Lewis* rats, Male	mTOR- dependent	Rapamycin	Not investigated	Ki-67: ---		
Fouraschen [160] 2013	*C57BL/6* mice Male, 12–15w	mTOR- dependent	Rapamycin and Steroid dexamethasone	LC3-II: +++	BrdU: --- PCNA: --- LWRR **: ---		
Kawaguchi [27] 2013	*C57BL/6J* mice, Male, 6–8w	mTOR- dependent	Temsirolimus	Not investigated	LBWR *: --- PCNA: ---		
Shi [25] 2018	*Balb/c* mice, 6–8w	mTOR- dependent	Rapamycin	LC3-II: +++	LBWR *: --- PCNA: ---		
			3-Methyladenine;			LC3-II: ---	LBWR *: +++ PCNA: +++
			*ASPP2*-haploinsufficient			LC3-II: --- p62: +++	LBWR *: +++ PCNA: +++

+++: Increase; ---: Decrease. * LBWR: Liver to body weight ratio. ** LWRR: Liver weight recovery rate. ^×^ used for estimating the capacity of LPCs differentiated into hepatocytes.

## Abbreviations

AMPK	AMP-activated protein kinase
AMP	Adenosine monophosphate
ASPP2	Apoptosis-stimulating protein two of p53
ATP	Adenosine triphosphate
Atg5	Autophagy related 5 protein
Atg7	Autophagy related 7 protein
Atg13	Autophagy related 13 protein
Bcl2	B-cell CLL/lymphoma 2
BrdU	5-Bromo-2′-Deoxyuridine
CDE	Choline-Deficient, Ethionine-supplemented
CDK	Cyclin-dependent kinase
CXCL7	Chemokine (C-X-C motif) ligand 7
C/EBP	CCAAT enhancer binding protein
DAPK-1	Death-associated protein kinase 1
DNA	Deoxyribonucleic Acid
EGF	Epidermal growth factor
EGFR	Epidermal growth factor receptor
ER	Endoplasmic reticulum
ERK1/2	Extracellular signal-regulated kinase 1/2
FFAs	Free fatty acids
GSK-3β	Glycogen synthase kinase 3 beta
HCs	Hepatocytes
HEK	Human embryonic kidney
HGF	Hepatocyte growth factor
HSC	Hepatic stellate cells
HSCs	Hepatic stem cells
IL-1	Interleukin 1
IL-6	Interleukin 6
IL-6R	Interleukin 6 receptor
IMPase	Inositol monophosphatase
Ins	Inositol
IPMK	Inositol Polyphosphate Multikinase
IRI	Ischemia-reperfusion injury
KD	Knockdown
KO	Knockout
LBWR	Liver to body weight ratio
LC3	Microtubule-associated protein 1A/1B-light chain 3
LKB1	Liver kinase B1
LPCs	Liver progenitor cells
LPS	Lipopolysaccharide
LSCs	Liver stem cells
LWRR	Liver weight recovery rate
MAPKs	Mitogen-activated protein kinases
MEFs	Mouse Embryonic Fibroblasts
mTOR	mammalian Target of Rapamycin
mTORC1	mammalian Target of Rapamycin complex 1
mTORC2	mammalian Target of Rapamycin complex 2
NADH	Nicotinamide adenine dinucleotide
NAFLD	Non-alcoholic fatty liver disease
NF-kB	Nuclear factor kappa B
NPC1L1	Niemann-Pick C1-Like 1
OCs	Oval cells
PCs	Progenitor cells
PCNA	Proliferating cell nuclear antigen
PDK1	Phosphoinositide dependent kinase 1
PH	Partial hepatectomy
PIP3	Phosphatidylinositol-3,4,5-triphosphate
PI3K	Phosphatidylinositol 3-kinase
PVL	Portal vein ligation
Rb	Retinoblastoma
Rheb	Ras homolog enriched in brain
ROS	Reactive oxygen species
SD	Sprague Dawley
S6K1	p70S6 kinase 1
SIRT1	Silent information regulator 1
Ski	Sloan-Kettering Institute
SnoN	Ski-related novel protein N
STAT-3	Signal transducer and activator of transcription 3
TFEB	Transcription Factor EB
TGF-α	Transforming growth factor alpha
TGF-β	Transforming growth factor beta
TNF-α	Tumor necrosis factor alpha
TNFR-1	Tumor necrosis factor receptor 1
TSC1/2	Tuberous sclerosis complex 1/2
ULK1	Unc-51 like autophagy activating kinase 1
3-MA	3-methyladenine
4E-BP	eIF4E binding protein
